# Vehicular emission inventory of criteria pollutants in Delhi

**DOI:** 10.1186/2193-1801-2-216

**Published:** 2013-05-10

**Authors:** Pramila Goyal, Dhirendra Mishra, Anikender Kumar

**Affiliations:** Centre for Atmospheric Sciences, Indian Institute of Technology Delhi, Hauz Khas, New Delhi, 110016 India

**Keywords:** Vehicular emission, IVE model, Pollutants, Delhi

## Abstract

The rapid urbanization in Delhi has resulted in a tremendous increase in the number of motor vehicles with the increase in population and urban mobilization. The vehicular traffic is now recognized as one of the main sources of air pollution in Delhi and has noticeable impact on air quality. The emission of criteria pollutants namely Carbon Monoxide (CO), Nitrogen Oxide (NO_x_) and Particulate Matter (PM) due to vehicles is estimated through the International Vehicle Emission (IVE) model, which includes the different driving modes of vehicles and meteorological parameters. The estimated emissions of Carbon Monoxide (CO), Nitrogen Oxides (NO_x_) and Particulate Matter (PM) due to different types of vehicles in the year 2008–09 are found to be 509, 194 and 15 tons/day respectively. The diurnal variation of emissions of air pollutants shows two peaks, which are fortunately matching with the morning and evening office hours. The emissions of CO and NO_x_ due to personal cars (PCs) are found to be about 34% and 50% respectively, and the emission of CO due to 2 W (2- Wheeler) is about 61%. Similarly, the Heavy Commercial Vehicles (HCVs) are contributing PM about 92%. The analysis of fuel-wise emission of pollutants reveals that CO is mainly contributed by petrol, and NOx and PM are contributed by diesel. It is also noticeable that CO, NO_x_ and PM emissions at ITO, one of the busiest traffic intersections of Delhi, are approximately 15, 6 and 0.5 tons/day respectively, which are found to be the maximum followed by Kashmiri Gate (ISBT), Nizamuddin etc. The present vehicular emissions inventory has been compared quantitatively with previous studies of Delhi. The present vehicular emission inventory has also validated using US environmental protection agency’s (USEPA’s) AERMOD model with observed concentration at different locations in Delhi. However, the present study shows that the air quality of Delhi has been degraded due to high level emissions of criteria pollutants.

## Introduction

Delhi is spread over an area of 1,484 km^2^ (573 sq mi), of which 783 km^2^ (302 sq mi) is designated rural and 700 km^2^ (270 sq mi) urban. The city's population is increasing rapidly with a consequent increase in the number of vehicles without a commensurate increase in road length. The number of vehicles registered in Delhi has already crossed 6 million and a sizeable vehicular traffic joins Delhi roads from the neighboring states (DSH 2010). It has been observed that vehicles alone contribute about 64% of the pollution in Delhi while other sources like power plants, industries, and domestic contribute 16%, 12% and 8% respectively (MoEF 1997). The maximum contribution of air pollution is growing rapidly from vehicular sources (Mitra and Sharma 2002). The main air pollutants emitted from these sources are Carbon monoxide (CO), Oxides of nitrogen (NOx) and Particulate Matter (PM). Some of them have been found to be much beyond the permissible levels governed by the Central Pollution Control Board (CPCB). The increasing levels of air pollutants are responsible for higher incidence rate of respiratory diseases, cancer, and heart diseases (Peters et al. 1997). Delhi’s degraded air quality is held responsible for about 18,600 premature deaths per year (TERI 2001).

**Figure 1:**
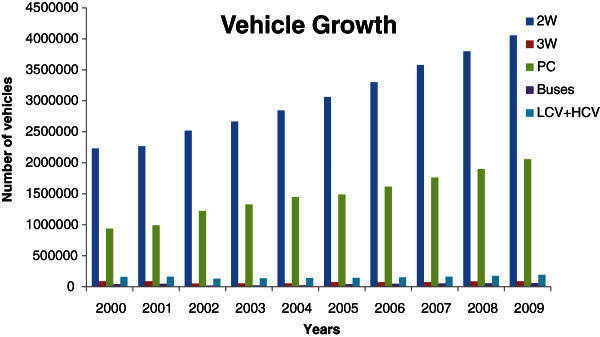
**Vehicle growth of Delhi.**

The number of vehicles per kilometer of road in Delhi has gone up from 128 to 191 between 2003 and 2009. This, despite the fact that the total available road space has increased from 30,698 lane kilometers in 2003 to 31,373 lane kilometer in 2009. The vehicle population growth in Delhi is sharply increased by an average annual rate 7.40% for private vehicles and 9.15% for commercial vehicles causing severe transportation and environmental problems (GNCTD 2010). It has been found that irrespective of road classes, about 30% of time, vehicles travel below 20 km/h speed. However, Delhi is at the position of the world’s 5^th^ worst city in traffic jam point of view (The Economic Times 2010). According to DSH (2010) report, the year wise vehicles growths are shown below in Figure 1.

There are several models used for estimation of vehicular emissions. The USEPA MOBILE model is one of them, which has been widely used. But in this model, the emission factors are conventionally based on the model years of US vehicle type and they are tested only on U.S. fuels and driving cycles, which can lead to incorrect emission values in India. So this model cannot be used for Delhi. One another vehicular emission model COPERT, which is widely used throughout Europe. However, this model cannot be used in Delhi as the emission factors used in this model are designed to be applied to average speeds for different road types. There are many more emission models such as LEAP (Long-range Energy Alternative Planning), VAPIS (Vehicle Air Pollution Information System) and Spreadsheet etc. But these models do not consider the variations in the local environment such as driving behaviors and traffic management and also assume that lower vehicle speeds increase emissions. In view of the above discussion, IVE model has been chosen for emission estimations. This model has been applied in several cities worldwide including Beijing and Shanghai, China (Nicole et al. 2005). The basic features of IVE model are based on (1) different modes of driving; (2) meteorological variables; and (3) emission factors of different pollutants with respect to different fueled vehicles.

The vehicle and fuel systems have to be addressed as a whole and jointly optimized in order to achieve significant reduction in emission. It was in 1996, the Ministry of Environment and Forests (MoEF), India, formally notified fuel specifications. In place of phase-wise up-gradation of fuel specifications, there appears to be a region-wise introduction of fuels of particular specifications. The high levels of pollution have necessitated eliminating leaded petrol throughout the country. To address the high pollution in 4 metro cities, 0.05% low sulfur diesel has been introduced since 2000–2001.

## Methodology

An emission inventory of main criteria pollutants CO, NO_x_ and PM due to vehicles in the year 2008–09 over the central part of Delhi has been developed as follows:

(i) A domain of the study area 26 km × 30 km (~780 km^2^ area) in central Delhi has been selected.
(ii) The numbers of vehicles monitored by Central Road Research Institute (CRRI) at 27 locations on different types of roads in the year 2008–09 has been used. It is noticeable that during the monitoring hours, there were no unusual conditions such as major processions, VIP visits, or other activities, which could induce abnormal traffic characteristics in the selected grids during the survey.
(iii) The diurnal variations of vehicular movement are observed at major traffic intersections.
(iv) An emission inventory of each type of vehicle with respect to CO, NOx and PM has been made individually.
(v) The diurnal variations of emissions during a study of start-up and running modes of different fueled vehicles have been estimated through IVE model.

**Figure 2:**
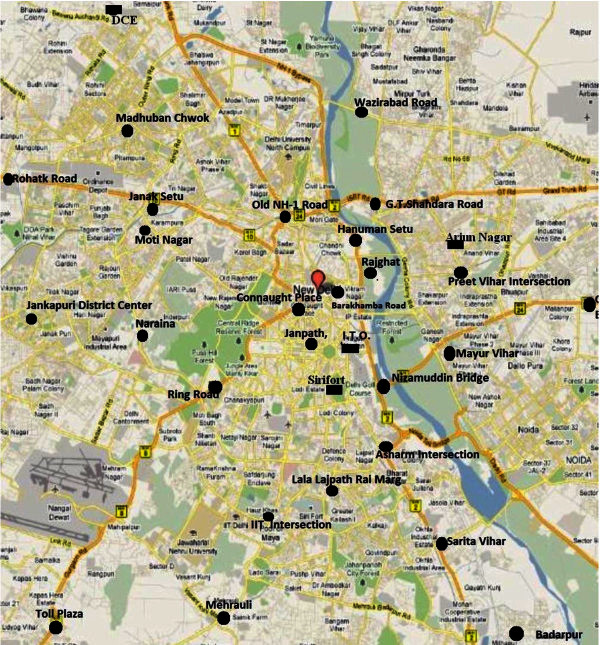
**Delhi study area (CRRI monitored station (●) and CPCB monitoring station (**^**▄▄**^**)).** (Source- http://www.mapmyindia.com).

The study area made over the map of Delhi has been shown in Figure 2, which has been divided into 195 grids of 2 km × 2 km size. IVE model is used to estimate the emissions due to vehicles in each grid of the study area. The monitoring stations of CRRI and CPCB over Delhi are also shown in Figure 2.

The input data of the model is prepared according to the format of the model, which are fleeting characteristics, vehicular activity and emission factors based on local conditions. A fleet file contains the base emission factor adjustment by technology and pollutants. The basis of the emission prediction process of the IVE model begins with a base emission rate and a series of correction factors, which are applied to estimate the amount of pollutants from a variety of vehicle types. On-road fleet characteristics are one of the important factors to estimate emissions. A vehicle activity files mainly requires the information of location/time, temperature, road grade, gasoline information, diesel information and driving pattern distribution. While vehicle emission rate files contain the information of base emission factor and correction factors. There are three critical components that are used in the IVE model to create accurate emissions inventories: (1) Vehicle emission rates (Base Emission Factor and Correction Factors); (2) Vehicle activity (Location Input Data); and (3) Vehicle.

The basis of the emission prediction process of the IVE model is to apply a base emission rate with a series of correction factors to estimate the amount of pollution from a variety of vehicles. Equation (1) estimates the adjusted emission rate by multiplying the basic emission rate by each correction factor.1

where, Q_[t]_: adjusted emission rate for each technology (start (g) or running (g/km); B_[t]_: base emission rate for each technology (start (g) or running (g/km). The correction factors used in equation 1 are categorized into several categories as local, fuel quality and power & driving variables. The local variables are ambient temperature, ambient humidity and altitude. The fuel quality variables are gasoline and diesel. The power & driving variables are road grade and air conditioning usage (IVEM, 2008). Equation (1) uses correction factors for eight parameters. Because of the lack of location specific data, an adjustment factor to the base emission rate “K_(Base)[t]_” has been taken equal to 1 that is the default value for IVE model and the fuel quality “K_(Fuel)[t]_”, inspection/maintenance correction factor “K_(IM)[t]_”, country correction factor “K_(Cntry)[t]_” and driving pattern “K_[dt]_;” correction factor are taken from IVEM BERCF 2008 and IVEM Correction Factor Data 2008. In addition to this, values for temperature “K_(Temp)[t]_”, humidity “K_(Hmdt)[t]_”, altitude “K_(Alt)[t]_”, and base emission rate “B[t]” have been taken from the IVE model dataset (IVEM Correction Factor Data 2008).

A data collection methodology has been designed to collect a large amount of data with the help of the resources typically available in Delhi. The hourly number of vehicles monitored at 27 different locations in Delhi by CRRI, have been collected. The roads are chosen in the representative of residential streets, arterials and freeways.

AERMOD is the new dispersion model, proposed for use in regulatory applications, in the United States. AERMOD is intended to replace the USEPA regulatory model, ISCST3. The U.S. EPA has evaluated AERMOD using several field databases. The AERMOD atmospheric dispersion modelling system is an integrated system that includes- 1) A steady-state dispersion model designed for short-range (up to 50 kilometers) dispersion of air pollutant emissions from stationary industrial sources. 2) A meteorological data preprocessor (AERMET) that accepts surface meteorological data, upper air soundings, and optionally, data from on-site instrument towers. AERMOD requires hourly surface and upper air meteorological observations for simulating the pollutant dispersion (USEPA 2004). The major purpose of AERMET is to calculate boundary layer parameters for use by AERMOD. The meteorological preprocessor of AERMOD, known as AERMET calculates boundary layer parameters, viz. frictional velocity, Monin–Obukhov length, convective velocity scale, temperature scale, mixing height, surface heat flux by using local surface characteristics in the form of surface roughness and Bowen ratio in combination with standard meteorological observations (wind speed, wind direction, temperature and cloud cover). These parameters are then passed through an interface present in AERMOD to calculate vertical profiles of wind speed, lateral and vertical turbulent fluctuations, and the potential temperature gradient. The hourly surface meteorological observations have been acquired from Indian Meteorological Department (IMD), Delhi and twice daily upper air soundings have been acquired from university of Wyoming’s upper air sounding.

## Results and discussions

The total emissions of CO, NO_x_ and PM from different types of vehicles over the study area of Delhi are found to be approximately 509, 194 and 15 tons/day respectively during the year 2008–09 and are shown in Table 1, which reveals that 2 wheelers (2W) and personal cars (PCs) are the mainly emitting the CO and NO_x_ respectively, while heavy commercial vehicles (HCV) are mainly emitting PM.

**Table 1:** **Emissions (tons/day) from each type of vehicle**

Fleet	CO	NO_x_	PM
2 W	311.2	5.8	0.40
3 W	14.4	34.5	0.01
PC	173.5	98.2	0.12
Bus	4.5	11.8	0.01
LCV	6.7	3.2	0.60
HCV	3.26	40.2	13.48
Total	509.6	193.7	14.62

It is also observed that the emissions of air pollutants at various locations as ITO, Kashmiri Gate (ISBT), Nizamuddin, Shahzada bagh, Sirifort, Shahdara are in decreasing order. The estimated values of emission of CO, NOx, and PM at ITO are as 15, 6 and 0.5 tons/day respectively.

It is noticeable that the emissions of different criteria pollutants are varying differently into the different operating conditions of vehicles, e.g., CO emissions are found to be higher during idling and decelerating than cruising, the NOx and PM emissions are lower during idling and decelerating than cruising. Therefore, shutting down the engine and restarting it will result in reduced emissions compared to allowing it to idle. Thus, one can say that the longer the shutdown period, the greater the emission benefits.

**Figure 3:**
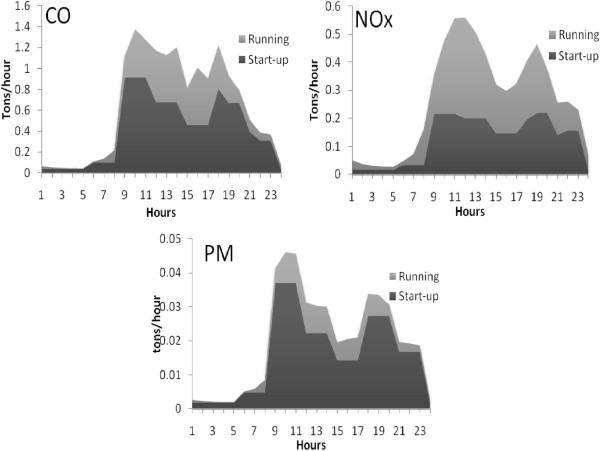
**Vehicle daily emission pattern at ITO.**

The diurnal variation of emissions of criteria pollutants CO, NOx and PM at ITO junction are shown in Figure 3. The two emission peaks during the day in each of these plots are evident, which indicate the peak traffic hours i.e., 8:00–10:00 in the morning and 17:00–19:00 in the evening, which are matched with starting and closing hours of most of the offices in Delhi. The emissions of air pollutants at peak traffic hours are 56% to 62%. This figure also shows the hourly variation of vehicular emissions of different air pollutants in start-up and running modes. The CO, NO_x_ and PM are emitted 86%, 27% and 71% respectively in start-up mode. The peaks of start-up emissions always appear between 8:00 am and 10:00 am, which is consistent with morning vehicle starts, and more than half of the starts are cold starts. (At start-up, especially during cold start, the temperature in the engine is low and fuel condensation is formed on the inner surface of the cylinder causing it to operate in the rich mode (high fuel-to-air ratio). This results in incomplete combustion of the fuel). Also, the catalysts are not operating when the temperature is not conductive to NOx formation. Therefore, the proportion of NOx emission is not as large as the other pollutants of vehicular emissions and the PM emission is always less than CO and NOx emissions.

**Figure 4:**
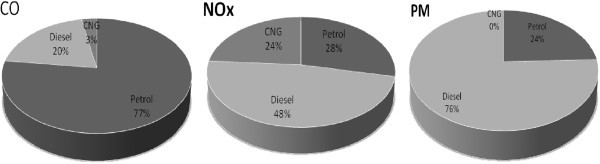
**Fuel wise emissions of pollutants from vehicles.**

The vehicle and fuel systems have to be addressed as a whole and jointly optimized in order to achieve significant reduction in emission. It was in 1996, the Ministry of Environment and Forests (MoEF), India formally notified fuel specifications. In place of phase-wise up-gradation of fuel specifications, there appears to be a region-wise introduction of fuels of particular specifications. The high levels of pollution have necessitated eliminating leaded petrol throughout the country. In order to address the high pollution in 4 metro cities, 0.05% sulfur petrol and CNG have been introduced since 2000–01 in place of diesel. After replacement of most diesel vehicles with CNG, the fuel wise emissions of air pollutants during the year 2008–09 are shown in Figure 4. The petrol, diesel and CNG vehicles emit CO as 77%, 20% and 3%, and NOx as 28%, 48% and 24% respectively. The diesel, petrol and CNG vehicles emit PM as 76%, 24% and 0% (negligible) respectively. On the basis of the above results, one can conclude that petrol and diesel vehicles are the main source of CO, NO_x_ and PM.

**Figure 5:**
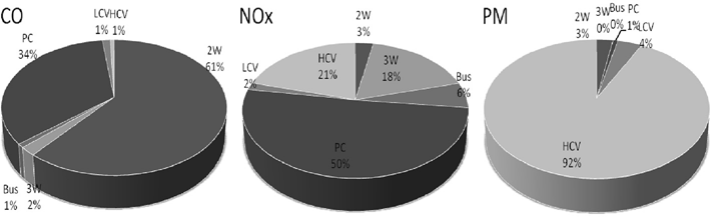
**Vehicle-wise emissions of criteria pollutants.**

The above discussion has also been supported by the vehicle-wise emission of air pollutants in Figure 5, which reveals that CO is contributed 61% by 2 W and 34% by PCs. It is obvious as 2 W are running on petrol. Similarly, the NOx is contributed 50% by PCs, which is in second position in most frequently running category of vehicles. Hence, in general 2 W and PCs are considered to be the main contributor of air pollutants in Delhi. However, 3 wheelers (3W) and buses are contributing very small or negligible amount of PM, since the CNG is used compressed natural gas in most of these vehicles.

### Evaluation of present estimated emissions

In general, it is always found to be difficult to evaluate the estimated emissions of air pollutants, since the real data on emissions are not available. However, in the present study (IIT study), the vehicular emissions of air pollutants are evaluated by comparing them with the estimated emissions of three previous studies by Kansal et al. (2011), Nagpure et al. (2011) and Gurjar et al. (2004) in Table 2, which shows a remarkable difference between the emissions of different studies. The first reason of this difference might be due to the consideration of the number of vehicles of different years and the second might be the use of different emission factors.

**Table 2:** **Comparative analysis of vehicular emissions (tons/day) from different studies**

Studies (Place-fuel)	CO	NO_x_	PM
Kansal et al. 2011 (ITO- all fuel) for year 2004-05	-	0.74	2.89
IIT study (ITO- all fuel) for the years 2008-09	14.89	5.87	0.49
Nagpure et al. 2011 (Delhi- petrol) for average values of years 1995-2005	216.45	9.85	-
IIT study (Delhi- petrol) for the year 2008-09	397.75	54.90	0.46
Gurjar et al. 2004 (Delhi- all fuel) for the year 2000	1210.47	363.98	76.52
IIT study (Delhi- all fuel) for the year 2008-09	509.62	193.46	14.59

The “IIT study” estimated the emissions of CO, NOx and PM due to all different fueled (petrol, diesel and CNG) vehicles over study area of Delhi for the year 2008–09 and emission factors by ARAI (2007). Kansal et al. (2011) studied emissions of NOx and PM at different locations due to all fueled vehicles for the year 2004–05 with the emission factor of Kandilkar and Ramachandran (2000). Nagpure et al. (2011) studied emissions of CO and NOx over Delhi due to petrol vehicles for the years 1995–2005 excluding 2 wheelers. Gurjar et al. (2004) estimated the emissions of the same pollutants due to different fueled vehicles for the year 2000 using emission factors of Kandilkar and Ramachandran (2000).

Kansal et al. (2011) estimated emissions by using number of all fueled vehicles of the year 2000–01 and emission factors of the study of Kandilkar and Ramachandran (2000), which are different than those used in the IIT study. The IIT study has used the number of all fueled vehicles of the year 2008–09 and emission factors by ARAI (2007) for estimating the emissions of CO, NO_x_ and PM. In order to make the comparison between two of the same year’s vehicles and emission factor, the IIT study has been made using the number of vehicles and emission factors for the year 2004–05. The emissions estimated by both the studies for NO_x_ and PM at different locations in Delhi are shown in Table 3, which shows a good agreement between both the emissions at different locations in Delhi.

**Table 3:** **Comparison of emissions between Kansal et al.**^2011^**and IIT study for the year 2004-05**

Locations	NO_x_Emissions (g/sec)			PM Emissions (g/sec)		
	Kansal et al.^2011^study for the year 2004-05	IIT study for the year 2004-05	% change	Kansal et al.^2011^study for the year 2004-05	IIT study for the year 2004-05	% change
ITO	33.40	40.96	22.63	8.60	10.95	27.27
Shazadabad	8.80	11.06	25.68	2.60	3.65	35.55
Janakpuri	6.40	8.52	30.00	2.60	3.34	28.39
Ashok Vihar	2.50	3.20	28.00	2.10	2.54	20.96
Shahdara	24.40	26.26	7.63	7.60	8.25	8.50
Sirifort	21.90	25.60	16.89	7.70	8.35	8.47
Nizamuddin	14.10	14.27	1.23	6.70	8.39	25.25

Similarly the IIT study has been made for the years 1995–2005. The comparison of average emissions for the same years of both the studies is shown in Table 4, which reflects a very small difference in both the values as 10–25%.

**Table 4:** **Comparison of emissions between Nagpure et al.**^2011^**and IIT study for the year 1995-2005**

Pollutants	Nagpure et al. 2011 for the average values of emissions of 1995-2005	IIT study for the average values of emissions of 1995-2005	% difference
CO	593 kg/year	652.67 kg/year	10.06%
NO_x_	27 kg/year	20.38 kg/year	24.44%

Same methodology has been adopted for comparing the emissions of IIT study and Gurjar et al. (2004). The IIT study estimated the emissions of the year 2000. The corresponding values are compared with the emissions of Gurjar's study and found that the values of emissions of both the studies for all three pollutants are in very good agreement as shown in Table 5. The difference is 6–17% in both of the studies.

**Table 5:** **Comparison of emissions between Gurjar et al.**^2004^**and IIT study for the year 2000**

Pollutants	Gurjar et al.^2004^for the year 2000	IIT study for the year 2000	% difference
CO	1210.47 tons/day	1054.83 tons/day	12.86%
NO_x_	363.98 tons/day	385.45 tons/day	5.89%
PM	76.52 tons/day	89.68 tons/day	17.19%

On the basis of the above discussion, it can be seen that emissions of IIT study for different years are very well matching with the emissions of the previous studies. Although, the methods used in all four are different. Finally it can be concluded that estimated emissions by IIT study are validated and can be considered for modeling studies and projection of future emissions. This study can also be used by policy makers and regulatory agencies for making some strategies for controlling the vehicular emissions in Delhi.

### Spatial distribution of vehicle emissions

**Figure 6:**
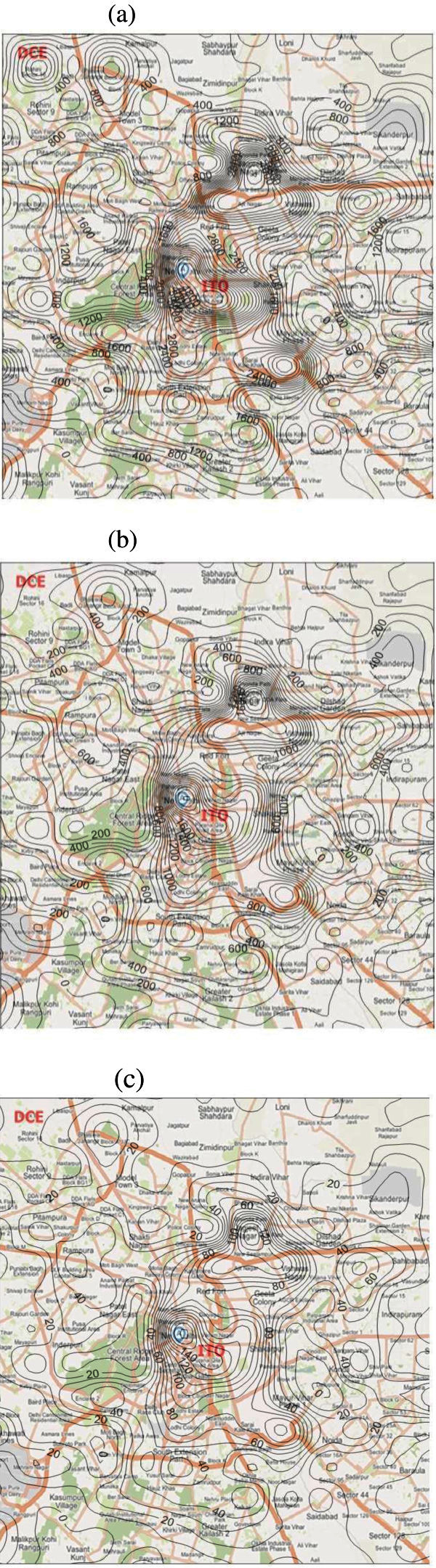
**Spatial distribution of vehicle emissions in Delhi in 2008–09 (t/yr) (a) CO (b) NOx (c) PM.**

The spatial (2 km × 2 km) emission inventories of CO, NOx and PM are shown in Figure 6. It shows that the spatial distributions of annual vehicle emissions. It can be observed that, although the spatial distribution trends of the three pollutants’ emissions are similar. The vehicular emission rate of CO and NOx are more in comparison to PM emissions. This may be because of the low vehicular emission factor of PM pollutant in comparison to CO and NOx pollutants.

### Simulation of the pollutant dispersion

The USEPA’s AERMOD model is a well accepted air pollution dispersion model worldwide. Currently, AERMOD is proposed the regulatory models for the Indian conditions. In this study, AERMOD model is adopted to simulate pollutant dispersion caused by motor vehicle emissions.

The dealing with meteorological data in AERMET, upper air data for Delhi cannot be used directly. The upper air data at 0:00 and 12:00 (UTC) switched in order to require by AERMET. The upper air data and surface air data for Delhi are received from the Atmospheric Science Department, University of Wyoming, Laramie, WY.

**Figure 7:**
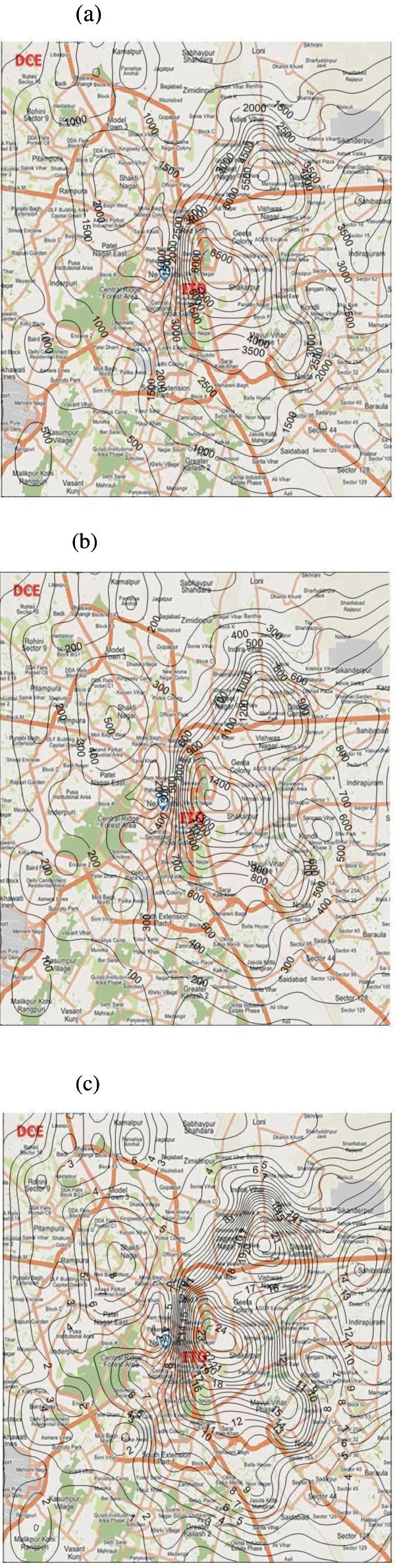
**Spatial distribution of annual-averaged emission concentrations caused by the motor vehicles in Delhi in 2008–09 (μg/m**^**3**^**) (a) CO (b) NOx (c) PM.**

The modeling results are shown in Figure 7. It is found that the spatial distributions of the three pollutant concentrations are similar with the spatial distributions of their emissions comparing with Figure 6. This is consistent with the fact that vehicular emissions usually affect their surrounding area, without considering the chemical reactions. It can also be observed that the annual concentrations of CO caused by on-road transportation are considerably high (the highest concentration is above approximately 6834 μg/m^3^), while the concentrations of PM caused by vehicular emissions are low (the highest concentration is about approximately 24 μg/m^3^). According to the National Ambient Air Quality Standards (NAAQS) in Delhi, the annual PM concentration standard is 60 μg/m^3^. Considering the annual PM concentration has exceeded the standard for the past several years, it is believed that motor vehicles are not only a major contributor of PM emissions in Delhi. Of course, the accurate estimation of concentration contributions from mobile source depends on the accurate estimation of other source emission inventories, mainly the stationary sources.

### Variations of predicted concentrations with observed concentrations

**Figure 8:**
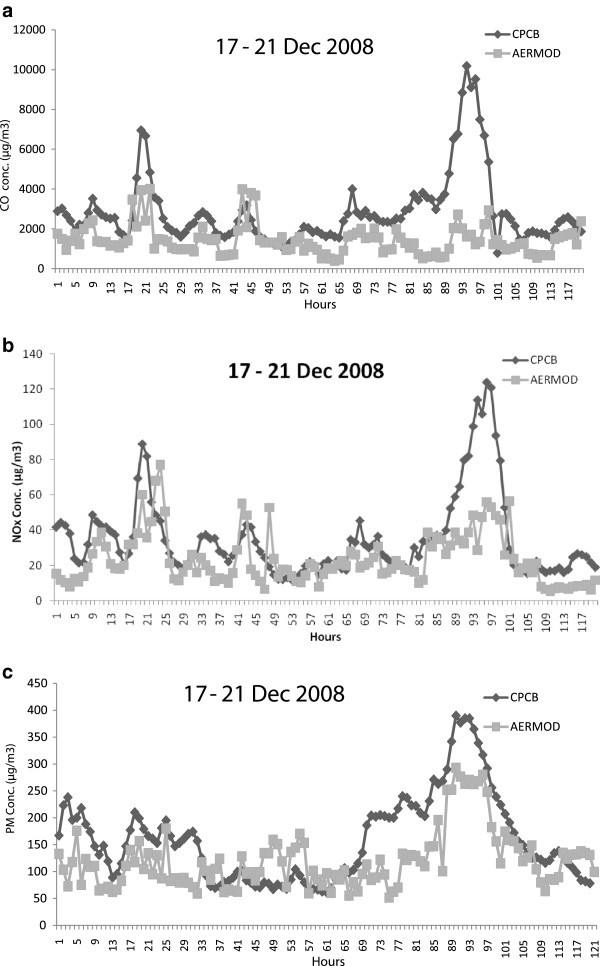
**Hourly variations of concentrations caused by the motor vehicles at ITO junction in Delhi during 17–23 December 2008 (μg/m**^**3**^**) (a) CO (b) NOx (c) PM.**

**Figure 9:**
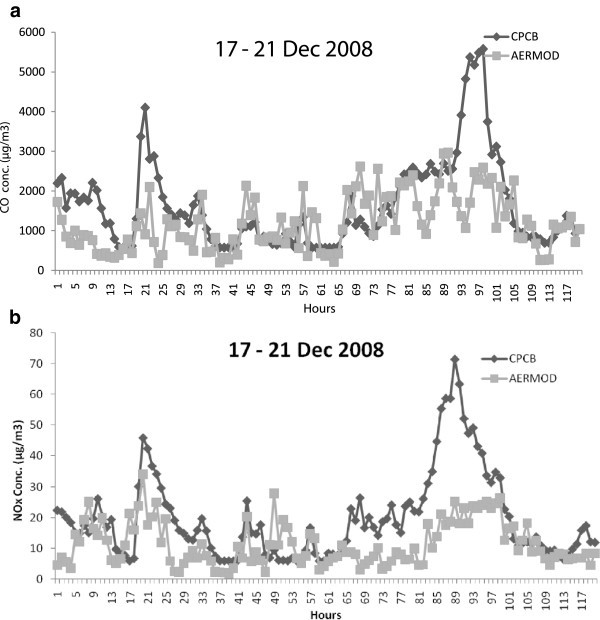
**Hourly variations of concentrations caused by the motor vehicles at DCE station in Delhi during 17–23 December 2008 (μg/m**^**3**^**) (a) CO (b) NOx.**

A comparative analysis of the AERMOD model predicted concentrations using the prepared emission inventory of criteria pollutants CO, NOx and PM against the observed concentration of that pollutant continuously monitored at ITO junction and DCE station. The winter month, December in the year 2008 has been chosen for the comparison of predicted with observed concentrations at both stations in Delhi. For the present study, 5 days have been chosen for representative month, December of the winter season to check the consistency of the concentrations with AERMOD. The dates chosen for the analysis are 17^th^ to 21^st^ December 2008. AERMOD has been executed to obtain the hourly CO, NOx and PM concentrations at ITO junction and then compared with the observed. Similarly the comparison of CO and NOx predicted concentrations from motor vehicles with the observed concentrations at DCE station also. Hourly variations of 5 days concentrations for Dec, 2008 at ITO junction and DCE station are shown in Figures 8 and 9 respectively . It is observed that the predicted concentrations of these criteria pollutants from motor vehicles are in good agreement with the observed concentrations from all sources. It is noticeable that the predicted concentrations of motor vehicles are approximately every hour lower than the observed concentrations of all sources at both stations in Delhi. It is also observed that the motor vehicle emissions are the main contributor in the observed concentrations of criteria pollutants. Again, the predicted concentrations of motor vehicles are positively correlated with the observed concentrations at ITO e.g. the values correlation coefficient for CO, NOx and PM pollutants are observed 0.33, 0.63 and 0.39 respectively. Similarly, the values of correlation coefficients for CO and NOx pollutants at DCE stations are 0.50 and 0.57 respectively.

## Conclusions

This paper develops vehicle emission inventory for Delhi, the capital of India, using for detailed study of emissions from vehicular sources. The purpose of this paper is to introduce a new grid based mobile source emission inventory using IVE model. The vehicle emissions of CO, NOx and PM are approximately 509, 194 and 15 tons per day respectively, in Delhi for the year 2008–09. The maximum emissions approximately 86%, 27% and 71% of the total emissions are emitted during the starting-up period for CO, NO_x_ and PM respectively. Petrol and diesel fuel vehicles are the main source of CO and PM pollutants respectively. CNG vehicles are one of the main contributors in NO_X_ emission. The emissions of the present study are evaluated with those of previous studies made by Kansal et al. (2011), Nagpure et al. (2011) and Gurjar et al. (2004), which reveals that the results of the present study are quite satisfactory and agreed well with previous studies. The spatial and temporal emission inventories of criteria pollutants from on-road vehicle emissions are justified with AERMOD analysis. The IIT emission inventory is superior to previous studies in the following manner as these three studies are based on number of vehicles, emission factor and vehicle kilometer travel. The IIT study calculates the emissions using IVE model, which also includes (1) different modes of driving; (2) meteorological variables in terms of correction factor; and (3) emission factors of different pollutants with respect to different type and fueled vehicles. However, the IIT emissions are seems to be comparable with earlier studies and are also validated through air quality model.

There are some drawbacks in the present emission inventory as the emission values of the present study would be improved if hourly monitored values of the number of all different types and different fueled vehicles at each grid point, i.e., at each 2 km distance would be available. The major uncertainty of the present study is the use of piecewise information concerning the emissions.
